# Gene-deficient mouse model established by CRISPR/Cas9 system reveals 15 reproductive organ-enriched genes dispensable for male fertility

**DOI:** 10.3389/fcell.2024.1411162

**Published:** 2024-05-21

**Authors:** Tuyen Thi Thanh Nguyen, Keizo Tokuhiro, Keisuke Shimada, Haoting Wang, Daisuke Mashiko, Shingo Tonai, Daiji Kiyozumi, Masahito Ikawa

**Affiliations:** ^1^ Department of Genome Editing, Institute of Biomedical Science, Kansai Medical University, Osaka, Japan; ^2^ Research Institute for Microbial Diseases, Osaka University, Osaka, Japan; ^3^ Graduate School of Pharmaceutical Sciences, Osaka University, Osaka, Japan; ^4^ Precursory Research for Embryonic Science and Technology (PRESTO), Japan Science and Technology Agency, Tokyo, Japan; ^5^ National Institute for Basic Biology, National Institutes of Natural Sciences, Okazaki, Japan; ^6^ Center for Infectious Disease Education and Research, Osaka University, Osaka, Japan; ^7^ The Institute of Medical Science, The University of Tokyo, Tokyo, Japan

**Keywords:** CRISPR/Cas9, knockout mice, male infertility, spermatozoa, testis

## Abstract

Since the advent of gene-targeting technology in embryonic stem cells, mice have become a primary model organism for investigating human gene function due to the striking genomic similarities between the two species. With the introduction of the CRISPR/Cas9 system for genome editing in mice, the pace of loss-of-function analysis has accelerated significantly. This has led to the identification of numerous genes that play crucial roles in male reproductive processes, including meiosis, chromatin condensation, flagellum formation in the testis, sperm maturation in the epididymis, and fertilization in the oviduct. Despite the advancements, the functions of many genes, particularly those enriched in male reproductive tissues, remain largely unknown. In our study, we focused on 15 genes and generated 13 gene-deficient mice [*4933411K16Rik*, *Adam* triple (*Adam20*, *Adam25*, and *Adam39*), *BC048671*, *Cfap68*, *Gm4846*, *Gm4984*, *Gm13570*, *Nt5c1b*, *Ppp1r42*, *Saxo4*, *Sh3d21*, *Spz1*, and *Tektl1*] to elucidate their roles in male fertility. Surprisingly, all 13 gene-deficient mice exhibited normal fertility in natural breeding experiments, indicating that these genes are not essential for male fertility. These findings have important implications as they may help prevent other research laboratories from duplicating efforts to generate knockout mice for genes that do not demonstrate an apparent phenotype related to male fertility. By shedding light on the dispensability of these genes, our study contributes to a more efficient allocation of research resources in the exploration of male reproductive biology.

## 1 Introduction

Approximately half of all infertility cases stem from male factors, affecting one in six couples globally, as reported by the World Health Organization (Agarwal et al., 2021). Male infertility, recognized as a complex clinical disorder with a wide range of phenotypic manifestations, has traditionally been categorized into pre-testicular, testicular, and post-testicular factors. However, a newer clinical classification now divides male infertility into four main categories: reproductive tract obstruction or dysfunction, hypothalamic-pituitary axis dysfunction, semen abnormalities, and reduced sperm quality and/or quantity (Tournaye et al., 2017; Krausz and Riera-Escamilla, 2018). Due to this multifaceted nature, diagnosing male infertility proves challenging, with the underlying causes of reproductive abnormalities remaining unclear in nearly half of cases.

In mammals, male fertility hinges on the consistent production of motile sperm capable of fertilizing an egg. Within testes, spermatogonial stem cells differentiate into spermatocytes, which then undergo meiosis to become haploid spermatids. These spermatids undergo significant changes, including acrosome formation, chromatin condensation, mitochondrial rearrangement, and flagellum formation, to develop into testicular spermatozoa (Yogo, 2022). Following this, sperm undergo additional maturation while traveling through the epididymis, acquiring the capability to reach the oviduct where they encounter ovulated eggs. Sperm passes through cumulus cells surrounding zona pellucida; only acrosome-reacted sperm can penetrate this layer. Finally, sperm fuse with the egg’s plasma membrane for fertilization (Ikawa et al., 2010). In testis, previous reports suggest that 1000–2000 germ cell-enriched genes may have the potential to regulate and facilitate these processes. (Schultz et al., 2003). Publications have discussed the expression of these genes, yet their *in vivo* functions in both humans and mice remain largely unknown. This is primarily attributed to the cost associated with knock-out techniques. However, ongoing exploration of whole gene functions in mice has been improving this situation (Miyata et al., 2016; Fujihara et al., 2018; Okabe, 2018).

Our research focuses on uncovering functions of genes enriched in the male reproductive system, elucidating their roles through detailed studies in gene-deficient mice (Miyata et al., 2016; Lu et al., 2019; Noda et al., 2019; Park et al., 2020; Sun et al., 2020). Here, we present loss-of-function analyses of 15 such genes (*4933411K16Rik*, *Adam20*, *Adam25*, *Adam39*, *BC048671*, *Cfap68*, *Gm4846*, *Gm4984*, *Gm13570*, *Nt5c1b*, *Ppp1r42*, *Saxo4*, *Sh3d21*, *Spz1*, and *Tektl1*), revealing that each is dispensable for male fertility.

## 2 Materials and methods

### 2.1 Animals

The animal experiments conducted in this study received approval from the Institutional Animal Care and Use Committees of Osaka University (Osaka, Japan) and Kansai Medical University (Osaka, Japan), adhering strictly to guidelines and regulations for animal experiments. B6D2F1 and ICR mice utilized in this research were purchased from CLEA Japan, Inc. (Tokyo, Japan), Japan SLC, Inc. (Shizuoka, Japan), or Shimizu Laboratory Supplies (Kyoto, Japan). In this study, all gene-modified mice generated on a hybrid genetic background of B6D2F1 and these mutant mice will be accessible to other researchers via either the RIKEN BioResource Research Center, Ibaraki, Japan, or the Center for Animal Resources and Development (CARD), Kumamoto University, Kumamoto, Japan (Supplementary Table S1).

### 2.2 Digital PCR

We generated digital PCRs (heatmaps) illustrating the average transcript per million values across a range of mouse and human reproductive and non-reproductive tissues, employing methodologies consistent with our previously published work (Robertson et al., 2020). In summary, sequences corresponding to various tissues were retrieved from Sequence Read Archives, subjected to trimming via TrimGalore, and aligned against the human genome (GRCh38) or mouse genome (GRCm38) utilizing HISAT2 (Kim et al., 2015). Gene expression levels in each tissue were quantified using Feature Counts, while RUVr was utilized for batch correction, eliminating unwanted variation (Risso et al., 2014). Differential gene expression between each non-reproductive tissue and reproductive tissue was determined using EdgeR (Robinson et al., 2010). To visualize the expression heatmap, we employed software accessible at https://orit.research.bcm.edu/MRGDv2.

### 2.3 Reverse transcription PCR (RT-PCR)

Total RNA from various tissues of wild-type mice were extracted using the TRIzol reagent (Thermo Fisher Scientific, Waltham, MA, United States) according to the standard procedure. Total RNA was reverse transcribed into cDNA using the SuperScript IV First-Strand Synthesis System (Thermo Fisher Scientific) and deoxyribonuclease (RT Grade) (Nippon Gene). PCR primers for each gene are listed in Supplementary Table S2.

### 2.4 Generation of KO mice with the CRISPR/Cas9 system

All gene-deficient mouse lines in this study were produced using the CRISPR/Cas9 system. CRISPRdirect software was used to find off-target sequences when we selected guide RNAs (Supplementary Table S3) (Naito et al., 2015). To prevent truncated protein expression and disrupt the functional role of proteins derived from mutated genes, we deliberately targeted the nearly complete coding regions of the gene locus. To generate gene-deficient mice, we performed electroporation using zygotes as described previously (Abbasi et al., 2018; Noda et al., 2019). The crRNA and tracrRNA (Integrated DNA Technologies, Coralville, IA, United States or Merck, Darmstadt, Germany) were mixed and hybridized by heating to 95°C and cooling to room temperature in the duplex buffer (Integrated DNA Technologies). The Cas9 protein (Thermo Fisher Scientific or Integrated DNA Technologies) was mixed with hybridized RNAs in Opti-MEM (Thermo Fisher Scientific). This solution was incubated at 37°C to generate the gRNA/Cas9 ribonucleoprotein (RNP) complex, and the resulting complex was electroporated into fertilized eggs using NEPA21 Super Electroporator (Nepagene, Chiba, Japan). The treated embryos that developed to the 2-cell stage were transplanted into the oviducts of pseudopregnant ICR females at 0.5 days after mating with vasectomized males. Pups (Founder 0) were obtained by natural or cesarean section and F0 mice that harbor each mutation are mated to wild-type B6D2F1 mice from a commercial source to produce heterozygous F1 offspring. Subsequently, F1 heterozygotes are interbred to produce F2 homozygous mutated offsprings. All experiments were conducted using F2 or F3-generation mice for analysis. Genotyping was performed by PCR using the primers listed in Supplementary Table S4. To examine the deleted region in the mutated allele of each line, we purified the amplified PCR products using the respective primers that detected the mutated allele. Subsequently, we subjected them to Sanger sequencing.

### 2.5 Morphological and histological analysis of testes

After measuring the whole body and testicular weight, testes were fixed in Bouin’s solution (Polysciences, Warrington, PA, United States or FUJIFILM Wako, Osaka, Japan), embedded in paraffin, sectioned at a thickness of 5 μm on a Microm HM325 microtome (Microm, Walldorf, Germany) or RM2125RT microtome (Leica, Nussloch, Germany), rehydrated, and treated with 1% periodic acid for 10 min. Schiff’s reagent (FUJIFILM Wako) was then applied for 20 min. The Periodic acid-Shiff staining sections were then stained with Mayer’s hematoxylin solution (FUJIFILM Wako). Testicular sections of *Adam* triple- and *Sh3d21*-deficient mice were stained with Mayer’s hematoxylin and EosinY solution (FUJIFILM Wako) before imaging. These sections were observed using an Olympus BX53 microscope equipped with an Olympus DP74 color camera (Olympus, Tokyo, Japan) or BZ-9000 (Keyence, Osaka, Japan).

### 2.6 Analysis of morphology and motility of spermatozoa

Spermatozoa from cauda epididymis were suspended in TYH medium (Muro et al., 2016), incubated at 37°C for either 10–30 min or 120 min, diluted, and then placed on MAS-coated glass slides (Matsunami Glass, Osaka, Japan) for morphology assessment using an Olympus BX53 microscope or placed in Leja glass chambers (imv Technologies, Orne, France) for sperm motility analysis using the CEROS II sperm analysis system (software version 1.5; Hamilton Thorne Biosciences, Beverly, MA, United States) or SMAS (DITECT, Tokyo, Japan).

### 2.7 Sperm-egg fusion assay

The fusion assay was performed as previously described (Inoue et al., 2005). Specifically, cauda epididymal spermatozoa were incubated in TYH drops for 2 h before the fusion assay. Unfertilized eggs were collected from hormone-treated females 14 h after hCG injection. To remove the ZP, eggs were treated with 1 mg/mL collagenase (038–22361, FUJIFILM Wako) for 30 min. After washing in TYH, eggs were stained with 1 μg/mL Hoechst 33,342 (DOJINDO, Kumamoto, Japan) for 10 min and washed repeatedly in TYH drops without Hoechst dye. For insemination, 2 × 10^5^ spermatozoa per mL were used. After incubation for 30 min, the eggs were fixed with 0.25% glutaraldehyde (FUJIFILM Wako), and we counted the percentage of fused eggs.

### 2.8 Fertility analysis of KO lines

Sexually mature control or null male mice were individually housed with two to three 8-week-old wild-type B6D2F1 female mice in the same cage for at least 8 weeks (except for *Adam20*, *Adam25*, *Adam39*-triple and *Sh3d21*-deficient mice). To ensure statistical validity, three or four males were tested for each knockout line. At the end of the mating period, male mice were removed from the cages, and the wild-type females were kept for an additional 3 weeks to allow for the delivery of their final litters. During the fertility test, the number of pups and copulation plugs were counted weekday mornings.

### 2.9 Statistical analyses

Statistical difference was determined using the Student’s t-test by Microsoft Office Excel (Microsoft Corporation, Redmond, WA, United States). Differences were considered statically significant if the *p* < 0.05. Data represent the mean ± standard deviation (s.d.).

## 3 Results

### 3.1 *In silico* expression analyses of 15 reproductive organ-enriched genes

**FIGURE 1:**
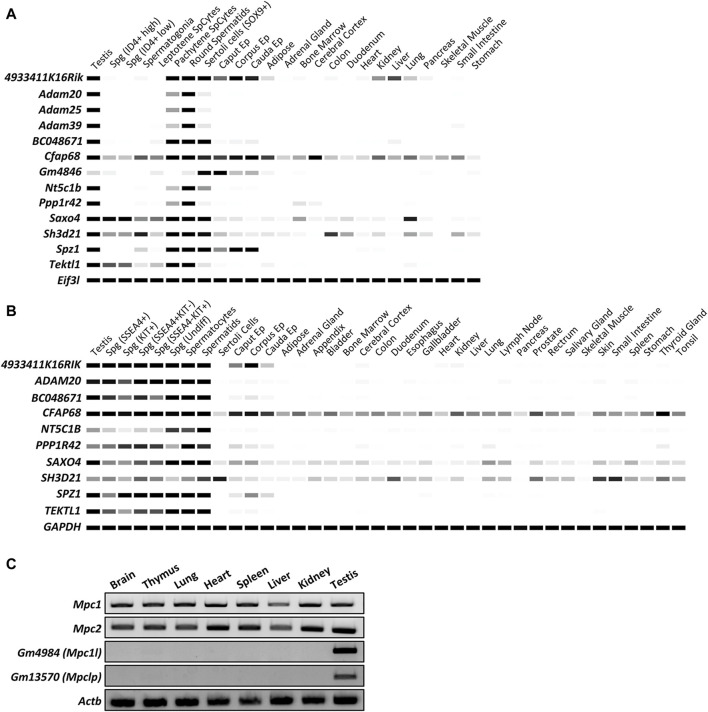
*In silico* analyses of the expression patterns of specific genes in multiple tissues and spermatogenic cells. **(A)** Digital PCR indicates the expression patterns of 13 genes of interest in mouse tissues. The average transcript per million (TPM) value per tissue per gene was generated from 77 published mouse RNA-seq data sets and 147 human RNA-seq data sets, respectively. Only *Cfap68* is detected slightly weak signals in ubiquitous tissues and highly expressed in testis and epididymis. The other 12 genes showed predominant or restricted expression in mouse testes or epididymides. White = 0 TPM, Black ≥30 TPM. *Eif3l* is used as an internal control. **(B)** Digital PCR indicates the expression patterns of 10 genes of interest in human tissues. White = 0 TPM, Black ≥30 TPM. *GAPDH* is used as an internal control. *ADAM25*, *ADAM39,* and *GM4846* are not found in human genomes. **(C)** RT-PCR indicates the expression patterns of *Mpc1*, *Mpc2*, *Gm4984,* and *Gm13570* in mouse tissues. *Actb* is used as an internal control.

We attempted to confirm the expression patterns of 13 genes of interest by digital PCR using Mammalian Reproductive Genetics Database V2 (Figures 1A,B). As shown in Figures 1A,B, all genes except *4933411K16Rik*, *Cfap68, Saxo4*, and *Sh3d21* demonstrated predominant expression in the testes or epididymides of mice and human. *4933411K16Rik*, *Cfap68*, *Saxo4*, and *Sh3d21* exhibited high expression levels in the testis and epididymis, with slightly weaker signals detected in somatic tissues. In a study by Benoît Vanderperre et al., additional members of the mitochondrial pyruvate carrier (MPC) family in placental mammals were identified using MPC1 and MPC2 protein sequences as input for analysis via the tblastn algorithm (Vanderperre et al., 2016). The additional MPC family genes, *Gm4984* and *Gm13570*, were not identified in these databases. We conducted reverse transcription-polymerase chain reaction (RT-PCR) analysis using mouse tissues, revealing that somatic-type genes encoding a mitochondrial pyruvate carrier protein, *Mpc1*, and *Mpc2*, were ubiquitously expressed. Meanwhile, *Gm4984* and *Gm13570*, predicted to be MPC family genes, exhibited specific expression in the testis (Figure 1C).

### 3.2 Production and phenotypic analyses of gene-deficient mice

Individual gene-deficient mouse lines were generated by CRISPR/Cas9 system, and phenotypic analyses were conducted to investigate the essentiality of these genes in male reproduction. The analyses of *Adam20*, *Adam25*, *Adam39*-triple, *Sh3d21*, *Gm4984*, and *Gm13570* deficient males will be described herein, whereas those of *4933411K16Rik*, *BC048671*, *Cfap68*, *Gm4846*, *Nt5c1b*, *Ppp1r42*, *Saxo4*, *Spz1*, *and Tektl1* deficient males are presented in Table 1 and Supplementary Figures S1–S3.

**TABLE 1:** Results of the fertility tests for the 13 mutant mouse lines. Each wild-type (WT) or mutant males were individually caged with WT female mice. NE indicates not examined.

Gene	Genotype	Average litter size ± SD	No. Of males	No. Of delivery	No. Of pups	No. Of plugs	Mating period (wks)
Wild type	-	8.9 ± 2.4	3	14	124	NE	15–21
*4933411K16Rik*	−880/-880	8.5 ± 3.0	3	20	170	28	8–16,18
*Adam25, Adam20, Adam39 (TKO)*	−78,899/−78,899	8.2 ± 1.9	4	20	164	NE	15–17
*BC048671*	−2,276/−2,276	9.3 ± 0.9	3	19	177	19	11
*Cfap68*	−1,857 + 3/−1,857 + 3	9.4 ± 1.6	3	23	217	24	8–16
*Gm4846*	−13,818/-13,818	9.4 ± 1.0	3	18	169	25	8
*Gm4984*	−1,558 + 2/−1,558 + 2	10.7 ± 1.9	3	23	245	28	8–16
*Gm13570*	−1,193 + 4/−1,193 + 4	8.9 ± 2.6	3	22	195	23	8–16
*Nt5c1b*	−18,291/−18,291	9.1 ± 1.8	3	19	173	21	8–16
*Ppp1r42*	−24,532/−24,532	8.0 ± 2.8	3	22	175	25	8–16,19
*Saxo4*	−7,755/−7,755	10.2 ± 2.6	3	20	204	24	11
*Sh3d21*	−12, 262/−12, 262	9.0 ± 2.2	4	14	123	NE	14–20
*Spz1*	−1,004 + 339/−1,004 + 339	8.5 ± 3.1	3	23	196	29	8–16
*Tektl1*	−4,648/−4,648	9.9 ± 2.3	3	21	208	22	11

**FIGURE 2:**
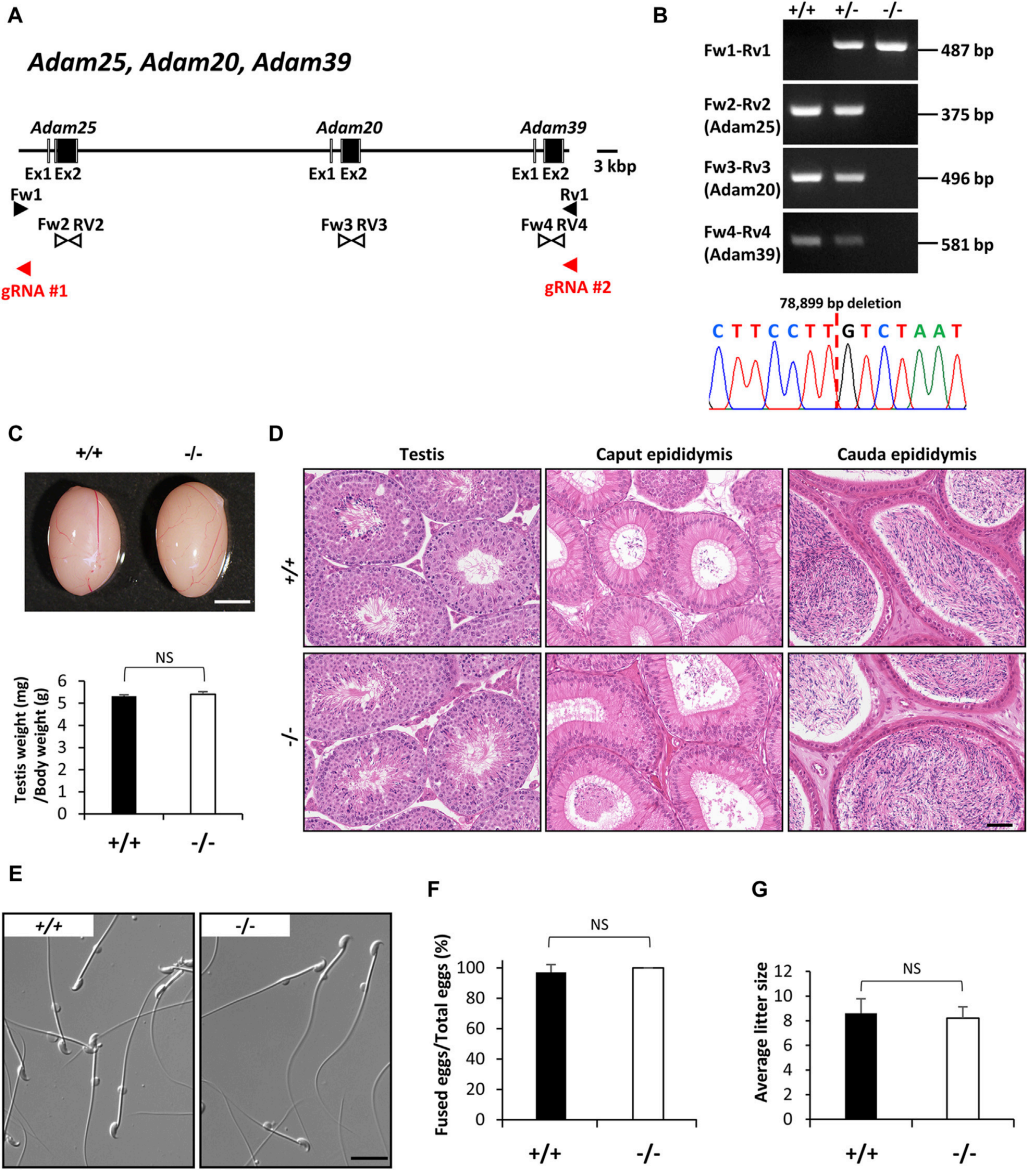
Phenotypic analysis of *Adam20, Adam25*, and *Adam39* triple (*Adam*-triple) -deficient male mice. **(A)** Genomic structure and knockout strategy of *Adam20*, *Adam25*, and *Adam39*. Two guide RNAs were designed to target the upstream of exon 1 of *Adam25* and downstream of exon 2 of *Adam39*. Eight primers (Fw1, 2, 3, 4 and Rv1, 2, 3, 4) were designed for genotyping the mutant mice. **(B)** Mutant and wild-type alleles were detected by genomic PCR using primer sets Fw1-Rv1 (mutant allele), Fw2-Rv2 (*Adam25* WT allele), Fw3-Rv3 (*Adam20* WT allele), and Fw4-Rv4 (*Adam39* WT allele). DNA sequence of the mutant allele by Sanger sequencing was shown in the lower panel. **(C)** Testis appearance and testis to body weight ratios of control (Wild type) and *Adam*-triple homozygous mutated mice. Scale bar = 3 mm. **(D)** Histological analysis of testes and epididymides in control and *Adam*-triple deficient mice. Scale bar = 50 μm. **(E)** Morphology of cauda epididymal spermatozoa in control and *Adam*-triple deficient mice. Scale bar = 20 μm. **(F)** Analysis of sperm and egg fusion ability. **(G)** Average litter size of control and *Adam* triple-deficient male mice. NS indicates not significant.

In 2017, Yan-Wei Sha et al., 2018 reported that the heterozygous variant in *ADAM20* mutation (NM_003814:exon2:c.641A>C:p.D214A) was found in an infertile male patient in China, who was concluded to be associated with sperm-egg fusion disorder. In mice, *Adam25* and *Adam39*, which are adjacent to the *Adam20* locus, are considered homologs of human ADAM20, as neither gene is identified in the human genome. These proteins might have a redundant role in mice. To assess the function of *Adam20* in mice, we established *Adam20*, *Adam25*, and *Adam39* triple deficient mice. *Adam20*, *Adam25,* and *Adam39* are positioned consecutively on the forward strand of mouse chromosome 8. These genes encode a single-pass type I membrane protein respectively and contain metalloproteinase, disintegrin, and EGF-like domains. Using two guide RNAs targeting the 5′ region of *Adam25* and the 3′ region of *Adam39*, we obtained mutant mice with a 78,899 bp deletion containing the coding region of the three genes (Figures 2A,B). Control and *Adam* triple null males displayed normal testicular appearance and comparable testis weights relative to body weights (Figure 2C). Histological analyses revealed no abnormalities in the testes and epididymides of control and *Adam* triple null males (Figure 2D). Additionally, both groups exhibited normal sperm morphology (Figure 2E). Despite reports in humans suggesting the importance of ADAM20 in sperm-egg fusion (Sha et al., 2018), our analysis using *Adam* triple deficient sperm revealed normal fusion ability compared to normal sperm (Figure 2F). Moreover, *Adam* triple deficient mice showed normal litter size mating with wild-type female mice (Figure 2G).

**FIGURE 3:**
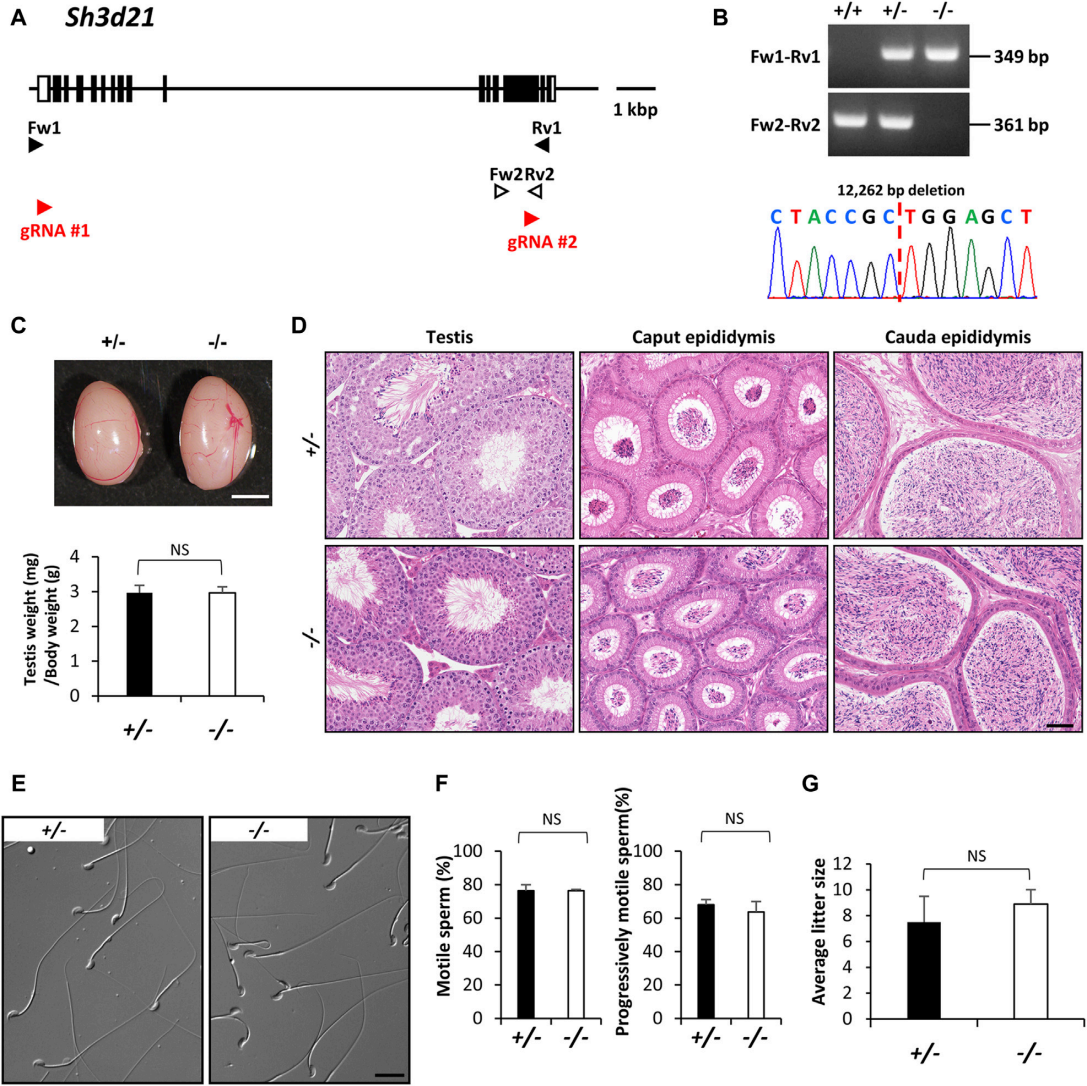
Phenotypic analysis of *Sh3d21* deficient male mice. **(A)** Genomic structure and knockout strategy of *Sh3d21*. Two guide RNAs were designed to target the first coding exon (Exon 1) and Exon 14. Four primers (Fw1, 2 and Rv1, 2) were designed for genotyping the mutant mice. **(B)** Mutant and wild-type alleles were detected by genomic PCR using primer sets Fw1-Rv1 (mutant allele) and Fw2-Rv2 (WT allele). DNA sequence and chromatograph of the mutant allele by Sanger sequencing were shown in the lower panel. **(C)** Testis appearance and testis to body weight ratios of *Sh3d21* heterozygous and homozygous mutated mice. Scale bar = 3 mm. **(D)** Histological analysis of testes and epididymides in control and *Sh3d21* deficient mice. Scale bar = 50 μm. **(E)** Morphology of cauda epididymal spermatozoa in control and *Sh3d21* deficient mice. Scale bar = 20 μm. **(F)** Proportion of motile sperm and progressively motile sperm after 120 min of incubation using computer-assisted sperm analysis (CASA). **(G)** Average litter size of control and *Sh3d21* deficient male mice. NS indicates not significant.

Protein phosphorylation plays a crucial role in germ cell development in mammals, spanning from spermatogonia to spermatids, and including key processes like sperm capacitation, motility, the acrosome reaction, and fertilization (Zhang et al., 2023). Several protein kinases, such as mitogen-activated protein kinases (MAPKs), aurora kinase C (AURKC), and Src family kinases (SFKs), are integral to various stages of spermatogenesis and are essential for mammalian fertility (Li et al., 2009; Coutton et al., 2015; Xiao et al., 2019). *SH3D21* is identified as a candidate gene in male infertility, linked to a maturation arrest of spermatogenesis in infertile patient groups (Stouffs et al., 2012). Additionally, it has been characterized as a protein that might have effects on other metabolic disorders and a sensitizer for gemcitabine in the treatment of pancreatic cancer (Hellwege et al., 2017; Masoudi et al., 2019; Tan et al., 2022). SH3D21, bearing the SRC homology 3 (SH3) domain, is found in signaling proteins and serves as a classical protein-protein interaction module. SH3 domain-containing proteins regulate a multitude of processes including cell-environment and cell-cell communication, cytoskeletal rearrangement, cell migration and growth, protein trafficking and degradation, and immune responses (Kaneko et al., 2008; Tatarova et al., 2012; Mayer, 2015). However, the specific functions of SH3D21 in male reproductive systems remain poorly understood. This protein is encoded by a gene located on the reverse strand of mouse chromosome 4. Similarly, *Sh3d21* deficient mice were produced using two guide RNAs targeting the region containing between exon 1 and exon 14, and we obtained mutant offspring bearing a 12,262 bp deletion were identified by genomic PCR and Sanger sequencing (Figures 3A,B). Phenotypic analyses demonstrated that *Sh3d21* null males displayed normal testicular appearance and weights (Figure 3C), as well as normal testicular and epididymal histology (Figure 3D). Moreover, they exhibited normal sperm morphology and motility (Figures 3E,F) and maintained normal *in vivo* fertility (Figure 3G).

**FIGURE 4:**
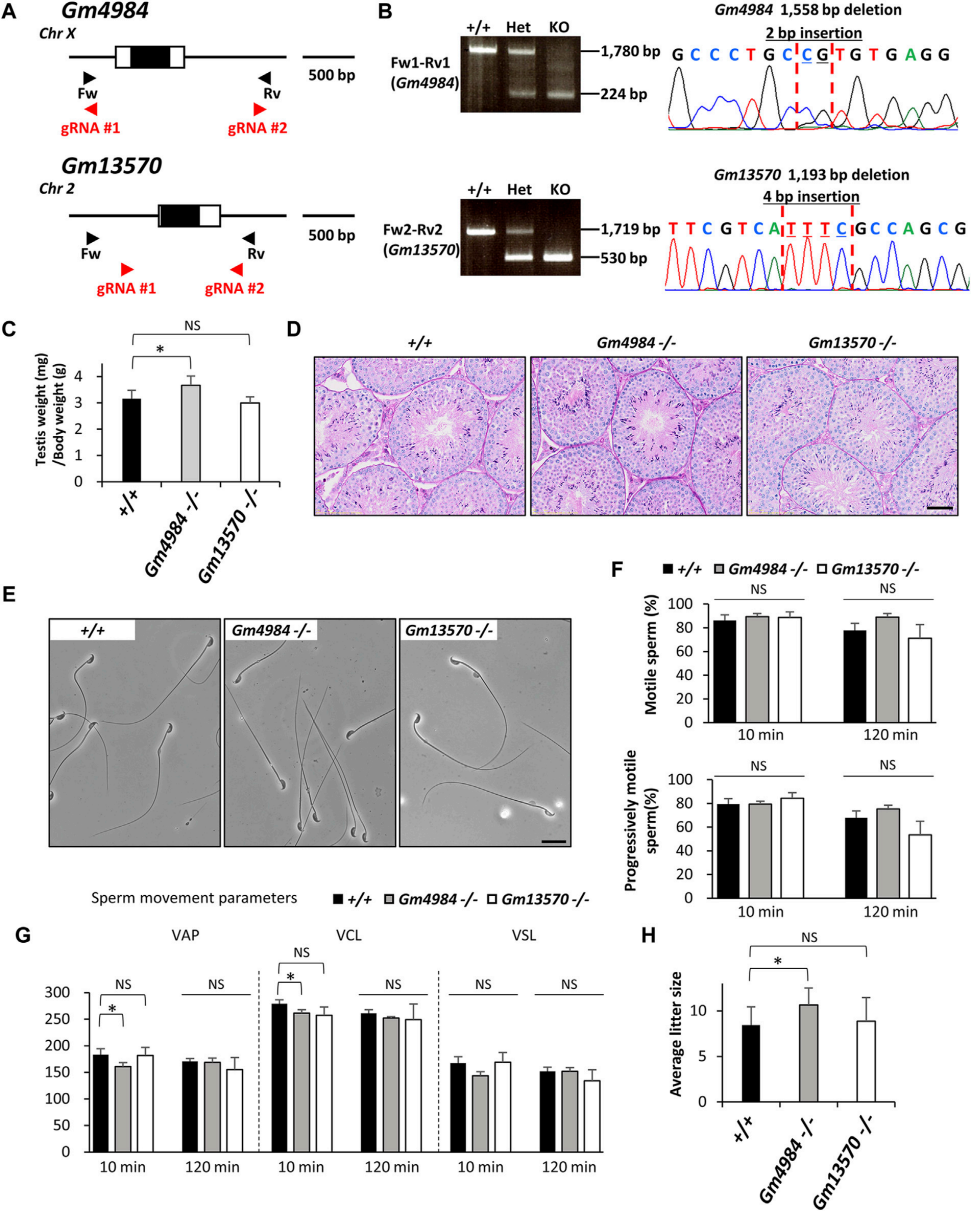
Phenotypic analysis of *Gm4984* and *Gm13570* deficient male mice. **(A)** Genomic structure and knockout strategy of *Gm4984* and *Gm13570*. Two guide RNAs were designed respectively to target the first coding exon (Exon 1) of both genes. Four primers (Fw1, 2 and Rv1, 2) were designed for genotyping the mutant mice. **(B)** Mutant and wild-type alleles were detected by genomic PCR using primer sets Fw1-Rv1 (wild-type and mutant allele of *Gm4984*) and Fw2-Rv2 (wild-type and mutant allele of *Gm13570*). DNA sequence and chromatograph of the mutant allele by Sanger sequencing were shown in the right panel. **(C)** Testis to body weight ratios of control, *Gm4984*, and *Gm13570* deficient mice. **(D)** Histological analysis of testes in control, *Gm4984*, and *Gm13570* deficient mice. Scale bar = 50 μm. **(E)** Morphology of cauda epididymal spermatozoa in control, *Gm4984*, and *Gm13570* deficient mice. Scale bar = 20 μm. **(F)** Proportion of motile sperm and progressively motile sperm after 10 and 120 min of incubation using computer-assisted sperm analysis (CASA). **(G)** Motility of cauda epididymal spermatozoa in control, *Gm4984*, and *Gm13570* deficient mice. Sperm motility and kinetic parameters were measured after incubation in TYH media for 10 and 120 min. VAP, average path velocity; VSL, straight-line velocity; VCL, curvilinear velocity. **(H)** Average litter size of control, *Gm4984*, and *Gm13570* deficient male mice. The reduction in litter size within each deficient line isn't notably significant when compared to the control group. * indicates *p* < 0.05 and NS indicates not significant.

Mitochondria play a vital role in cellular energy production, generating ATP and metabolites essential for cell survival and growth. Pyruvate, a key metabolite, serves as a major source of acetyl-CoA required for the tricarboxylic acid (TCA) cycle, a critical component of aerobic respiration (Martínez-Reyes and Chandel, 2020). Acetyl-CoA derived from pyruvate also contributes to synthesizing lipids and amino acids, supporting anabolic processes. Thus, understanding the molecular mechanisms governing pyruvate fate within cells is crucial for comprehending cellular metabolism regulation and overall cell function and fate. Central to this process is the transport of pyruvate from the cytosol to the mitochondrial matrix. It is believed that cytosolic pyruvate transport to the outer mitochondrial membrane via voltage-dependent anion channel/porin channels (Palmieri et al., 2011) and the inner mitochondrial membrane via a specific transporter called the mitochondrial pyruvate carrier (MPC) complexes (Bricker et al., 2012; Herzig et al., 2012). *Gm4984* (MPC1L-mitochondrial pyruvate carrier 1 like) and *Gm13570* (MPCLP- mitochondrial pyruvate carrier like protein) were identified as candidates of MPC1 family based on protein sequence similarity and are expressed at very high concentrations in mice, particularly in the mitochondria of adult reproductive tissues (Vanderperre et al., 2016). *Gm4984*, located as a single-exon gene on the forward strand of mouse chromosome X, and *Gm13570*, also a single-exon gene positioned on the reverse strand of mouse chromosome 2, are predicted to encode mitochondria-localized proteins with a role as pyruvate carriers. (Vanderperre et al., 2016) (Figure 4A). Using two guide RNAs in each gene that flanking the single exon, we introduced a 1,558 bp deletion (+2 bp insertion) into the locus of *Gm4984* and a 1,193 bp deletion (+4 bp insertion) into the locus of *Gm13570* (Figures 4A,B). *Gm4984* null mice displayed slightly higher ratio compare to control and *Gm13570* null mice displayed normal testis weights (+/+: 3.2 ± 0.3, *Gm4984* null: 3.7 ± 0.4, *Gm13570* null: 3.0 ± 0.3, Figure 4C). Both *Gm4984* null and *Gm13570* null mice displayed normal testicular histology (Figure 4D). Furthermore, both null mice exhibited normal sperm morphology (Figure 4E), with computer-assisted sperm analysis indicating comparable sperm motility and progressive motility (Figure 4F). In terms of sperm swimming velocity comparison between control and null males, there was a slight decrease in the VAP and VCL parameters in *Gm4984* null at the 10 min incubation. However, after 120 min of incubation, which induced capacitation, there were no significant differences observed in any parameter (Figure 4G). Importantly, both null mice maintained normal *in vivo* fertility and slightly increased the litter size in *Gm4984* null mice (+/+: 8.4 ± 2.0, *Gm4984* null: 10.7 ± 1.9, *Gm13570* null: 8.9 ± 2.6, Figure 4H).

### 3.3 Fertility results

Through housing the gene-deficient males individually with wild-type females, we discovered that these males were able to sire offspring with average litter sizes comparable to those sired by wild-type males (Table 1). Hence, all mutant males examined in this study displayed normal fertility.

## 4 Discussion

About 15 out of 100 couples have trouble getting pregnant. Roughly half of infertility cases are attributed to males and not fully understood, and there may be thousands of genes that can be harbored for this issue (Esteves, 2013; Lotti and Maggi, 2018). The application of the CRISPR-Cas9 system has helped clarify certain genes related to male reproduction (Miyata et al., 2016; Lu et al., 2019; Park et al., 2020; Sun et al., 2020). In this study, we continue utilizing CRISPR/Cas9 to analyze the role of these 15 genes in male reproduction. All of the genes under analysis exhibited higher expression levels in the human and mouse testes or epididymis. Despite the limited expression of some of these genes in other tissues, we did not observe any aberrant behavior or appearance in the mutant mice. This suggests that these genes may not be important for human male reproduction either.

The A Disintegrin and Metalloproteinases (ADAMs) belong to the metalloproteinase superfamily, which comprises a diverse group of multi-domain transmembrane and secreted proteins with varied biological functions such as inflammation and cancers, and reproductive functions (Cho, 2012; Arai et al., 2023). In 2017, Yan-Wei Sha et al., 2018 identified a heterozygous variant (NM_003814:exon2:c.641A>C:p.D214A) in *ADAM20* in an infertile male patient in China, associated with a sperm-egg fusion disorder. *Adam25* and *Adam39*, neighboring *Adam20* locus in mice, are considered human *ADAM20* homologs. To confirm *Adam20 in vivo* function in mammals, we developed triple deficient mice lacking *Adam20*, *Adam25*, and *Adam39*. Surprisingly, the *Adam* triple deficient mice showed normal spermatogenesis, sperm morphology, fusion ability, and *in vivo* fertility compared to control. In mice, many ADAM proteins that are specifically expressed in reproductive tissues were identified and remained unknown function in mammalian fertility (Cho, 2012). These proteins might work as a key molecule in the fusion ability of sperm or compensate for the function of ADAM20 in mice. The advancements in next-generation sequencing have revolutionized genome characterization, facilitating precise identification of human genome mutations that may underlie disease. However, validating whether gene deficiency or abnormalities cause the disease *in vivo* is essential. Utilizing gene-deficient mice models is a potent strategy for pinpointing disease-causing genes. These models are pivotal in unraveling critical functions, not only in reproductive biology but also in other areas.

Protein phosphorylation is pivotal in mammalian germ cell development, encompassing processes from spermatogonia to spermatids, including sperm capacitation, motility, the acrosome reaction, and fertilization (Zhang et al., 2023). *SH3D21* emerges as a candidate gene in male infertility, associated with spermatogenesis maturation arrest in infertile patients (Stouffs et al., 2012). However, its specific roles in male reproductive systems remain unclear. Digital PCR analyses indicate high expression of *Sh3d21* in the testis, implying potential significant roles during spermatogenesis. Surprisingly, *Sh3d21* deficient mice displayed normal spermatogenesis and *in vivo* fertility. The SRC homology 3 family encompasses approximately 300 annotated proteins containing SH3 domains in humans (Mayer, 2015), suggesting that other family genes enriched in the testis might compensate for the function of SH3D21.

*Gm4984* (MPC1L-mitochondrial pyruvate carrier 1 like) and *Gm13570* (MPCLP- mitochondrial pyruvate carrier like protein) were identified as MPC1 family candidates based on protein sequence similarity. They are highly expressed in mice, particularly in adult reproductive tissue mitochondria (Vanderperre et al., 2016). While GM4984 in humans and mice are highly conserved in the first 100 amino acids and are exclusively expressed in round and elongated haploid spermatids during their differentiation into mature sperm cells (Vanderperre et al., 2016), our observations did not reveal any abnormalities in the male reproductive system of both gene-deficient mice. Testicular germ cells and mature sperm possess MPC1 and MPC2, which are somatic types of mitochondrial pyruvate carrier complex subunits. It is plausible that the functions of these genes are sufficient to complete the fertilization process under natural conditions, despite the potential importance of GM4984 and GM13570 in the male reproductive system.

PPP1R42, localized at the basal body primary cilia in retinal epithelial cells, has been implicated in maintaining centrosome number. Inhibition of PPP1R42 expression results in increased centrosome number, leading to mis-segregation and cell death (DeVaul et al., 2013). *Cfap68* gene encodes cilia and flagella-associated protein 68, which is involved in the structural specialization of the sperm tail (Leung et al., 2023). SAXO4 protein is a member of the stabilizer of axonemal microtubules (SAXO) group which decorates sperm doublet microtubules and likely stabilizes the tubulin lattice during sperm movement. TEKTL1 (CCDC105) which is a tektin-like sperm-microtubule inner protein, has been described as a conserved, sperm-specific, tektin-like molecule that forms a filament in the cleft between protofilaments and binds several core- and sperm-microtubule inner proteins. It may help stabilize the outer junction which suggests that deletion of *Tektl1* may lead to fertility problems. These mice deficient in each protein showed normal spermatogenesis, sperm morphology, and *in vivo* fertility. Utilizing *in situ* cryoelectron tomography (Cryo-ET), three research groups have identified proteins associated with microtubule doublets (DMTs) and microtubule inner proteins (MIPs) within sperm flagella (Chen et al., 2023; Leung et al., 2023; Tai et al., 2023). Among these, *Tekt5* is identified as a gene expressed specifically in the testis and is expected that play essential roles in DMTs structure. Surprisingly, *Tekt5* null mice exhibit normal fertility despite an increased ratio of sperm with defective flagella (Chen et al., 2023). Cryo-ET analysis of *Tekt5* null sperm reveals low occupancies in 3-helix bundles and this analysis may reveal abnormalities in the flagellum of *Tektl1* null sperm. These findings suggest that while structural biology provides intricate insights into biomolecular architecture, functional studies are indispensable for unraveling their biological significance and contributions to physiological processes and diseases. This synergy between structural and functional approaches is vital for a comprehensive understanding of biology. Other similar proteins, CFAP proteins, SAXO proteins, and TEKTIN proteins were identified in sperm flagellar components in sea urchin and bovine and these proteins might have duplicate roles and compensate for the function of deficient proteins (Leung et al., 2023). While *Cfap68* exhibits weak expression in various tissues such as cerebral cortex, kidney, lung, and small intestine of mice, its deficiency does not seem to lead to obvious abnormalities or lethality. Delving deeper into the analysis of these tissues could potentially unveil additional functions of *Cfap68* beyond what is currently investigated in this study.

Similarly, we identified four other relatively unknown genes—*4933411K16Rik*, *BC048671*, *N15c1b*, and *Spz1* that are highly expressed in the testis and *Gm4846* specifically expressed in the epididymis (Hammarström et al., 1995; Hsu et al., 2001; Kiyozumi et al., 2020; Kiyozumi et al., 2023; Kiyozumi, 2024). Despite their significant expression patterns, our study revealed that these genes are not essential for male fertility, as no differences were observed between control and gene-deficient mice in terms of fertility.

In summary, our study utilized the CRISPR/Cas9 system to disrupt 15 genes enriched in reproductive tissues in mice. Through *in vivo* functional analysis of 13 established gene-deficient mouse lines, we demonstrated that none of these genes are essential for normal male fertility. These gene-deficient mouse lines will be deposited at public institutions in Japan, facilitating future research to explore overlapping gene functions in mammals and understand the mechanisms underlying male reproductive function. The evolution of the CRISPR/Cas9 system has enabled the creation of gene-deficient animals in various experimental species, offering valuable insights into reproductive biology (Lee et al., 2018). Furthermore, exploring proteins like acrosin may reveal significant implications in other animal species, driving forward our understanding of reproductive processes and unveiling novel perspectives (Hirose et al., 2020).

## Data Availability

The raw data supporting the conclusion of this article will be made available by the authors, without undue reservation.
