# Supplementary material for: Gene-deficient mouse model established by CRISPR/Cas9 system reveals 15 reproductive organ-enriched genes dispensable for male fertility
Source: Front Cell Dev Biol. 2024 May 21;12:1411162. doi: 10.3389/fcell.2024.1411162 (PMC11148293; doi:10.3389/fcell.2024.1411162)
Supplement: Supplementary file 3 [file Image1.PDF]

Supplementary Material

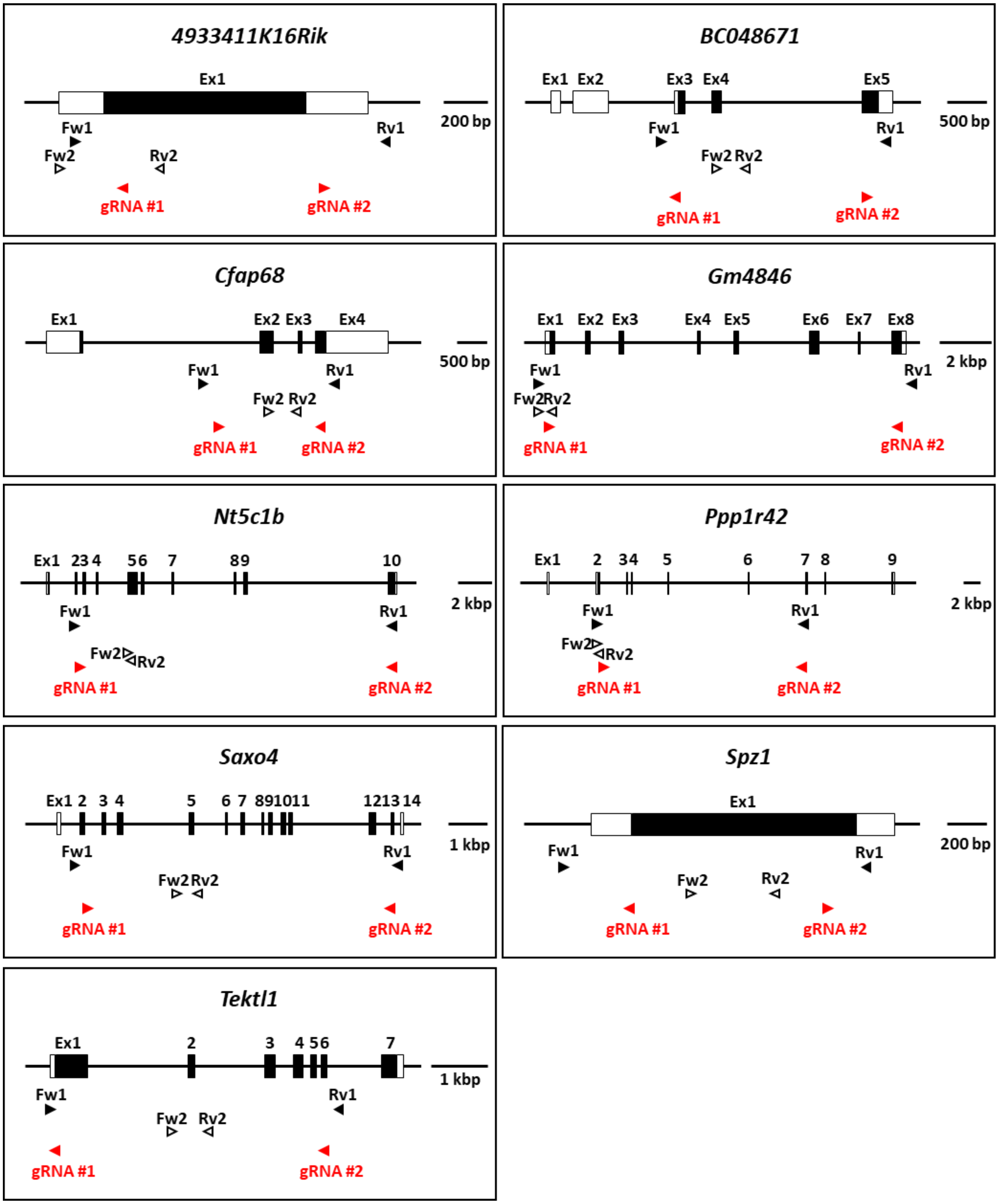

Supplementary Figure 1. Schematic representation of the genomic structure and the Knockout strategy in each line.

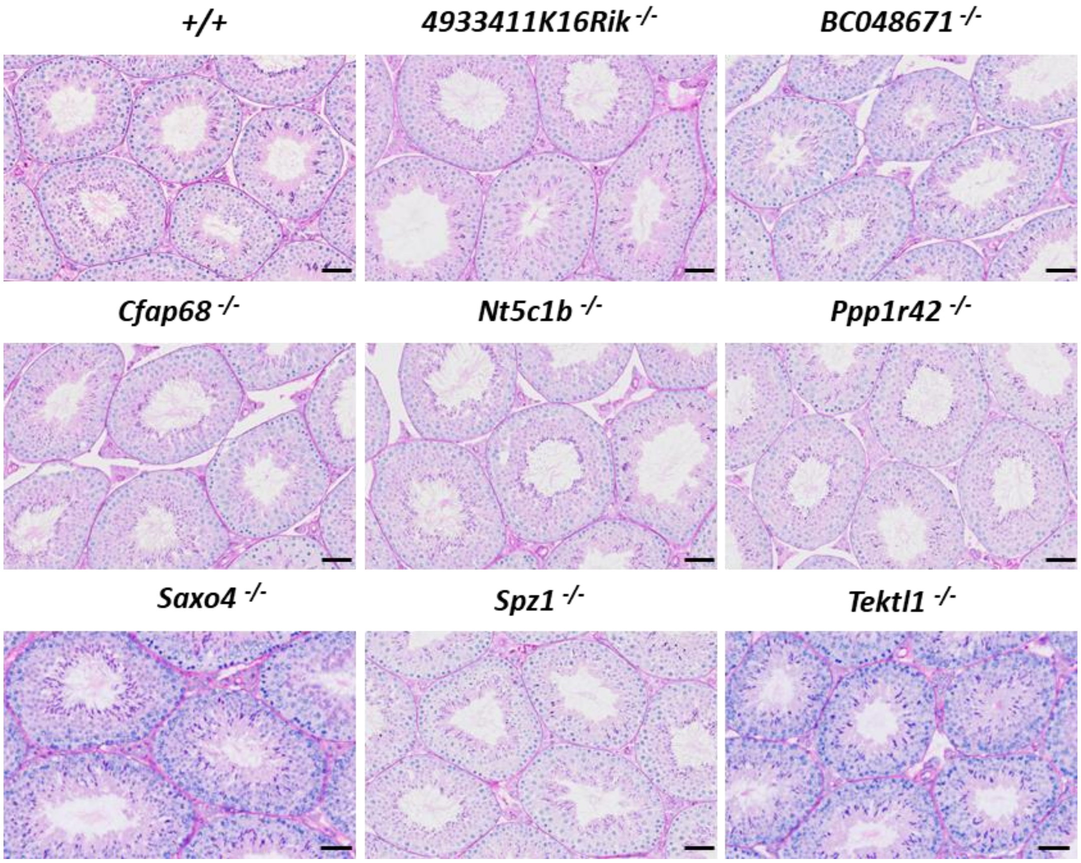

Supplementary Figure 2. Histological analysis of testes showed normal morphology in each line. We did not observe the testicular section of *Gm4846*-deficient mice. Scale bar=50 µm.

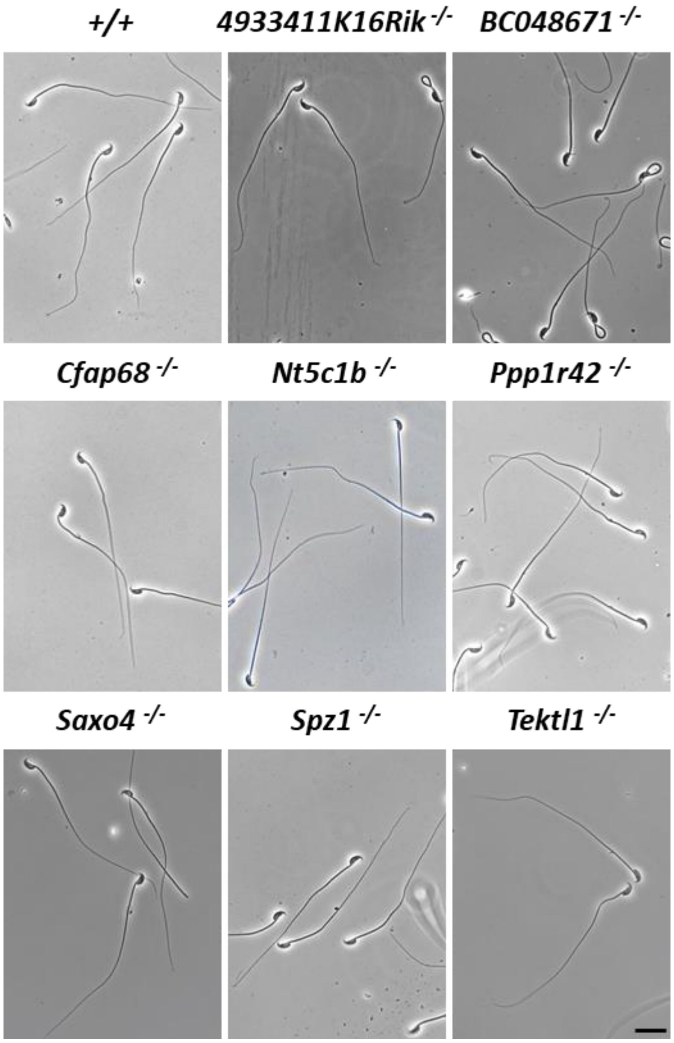

Supplementary Figure 3. All sperm isolated from cauda epididymis in each line showed normal morphology. We did not observe the epidydimal sperm of *Gm4846*-deficient mice. Scale bar=20 µm.

Supplementary Table 1. The 13 gene-deficient mouse lines in this study. The RBRC No. and CARD ID are available for the mouse lines that have been deposited as frozen sperm at Riken Bio Research Center and Center for Animal Resources and Development at Kumamoto University, respectively.

Gene symbol	Gene Name	RBRC No.	CARD ID
4933411K16Rik	RIKEN cDNA 4933411K16 gene	12244	3475
Adam20, *Adam25*, *Adam39* (TKO)	a disintegrin and metallopeptidase domain 20<br>ADAM metallopeptidase domain 25<br>a disintegrin and metallopeptidase domain 39	12068	NA
BC048671	cDNA sequence BC048671	12242	3473
Cfap68	cilia and flagella associated protein 68	12234	3465
Gm4846	predicted gene 4846	11027	2934
Gm4984	predicted pseudogene 4984	12209	3440
Gm13570	predicted gene 13570	12218	3449
Nt5c1b	5'-nucleotidase, cytosolic IB	12255	3486
Ppp1r42	protein phosphatase 1, regulatory subunit 42	12243	3474
Saxo4	stabilizer of axonemal microtubules 4	in process	in process
Sh3d21	SH3 domain containing 21	12066	NA
Spz1	spermatogenic leucine zipper 1	12256	3487
Tektl1	tektin like 1	in process	in process

Supplementary Table 2. Primer information for RT-PCR.

Gene symbol	Primer sequences	Amplified DNA products (bp)
Mpc1	Fw: GTCTAGGTAGCGGCTTCACC<br>Rv: GTGGGTTTAGGGACTCTCGG	521
Mpc2	Fw: ACCTACCACCGACTCATGGA<br>Rv: ATCCGAAACAGCTGAGAGGC	308
Gm4984 (*Mpc1l*)	Fw: AATGCGGAAAACGCTAGATT<br>Rv: CATCCCTTCCCCCAGTCATGTCATG	364
Gm13570 (*Mpclp*)	Fw: GTAGCGCTAATGCGGAAAAC<br>Rv: TGTCAAATTCACTTGGGACTC	527
Actb	Fw: CATCCGTAAAGACCTCTATGCCAAC<br>Rv: ATGGAGCCACCGATCCACA	171

Supplemental Table 3.

Two guide RNAs targeting the upstream and downstream regions of each gene were used to generate gene-deficient mice. The efficiency of embryo transplantation was represented by the number of total pups delivered by pseudo-pregnant mice divided by the number of total embryos used for oviduct transplantation. The efficiency of genome editing was determined by the number of pups carrying gene deletion divided by the number of pups subjected to genotyping. We performed Sanger sequencing analysis of the deletion allele and identified the deleted sequence information and DNA length in each gene-deficient line.

Supplemental Table 4.

The sequence information of primers and PCR conditions used for genotyping in each line.
